# Gait Kinematic Adaptations Following Schroth Therapy in Individuals with Adolescent Idiopathic Scoliosis

**DOI:** 10.3390/jcm15103661

**Published:** 2026-05-10

**Authors:** Hande Argunsah, Recep Buğra Sarıkaya, Tuğçe Yavuz, Akif Albayrak

**Affiliations:** 1 Department of Biomedical Engineering, Faculty of Engineering and Natural Sciences, Acibadem Mehmet Ali Aydinlar University, 34752 Istanbul, Turkey; recepbugra1@gmail.com; 2 Graduate School of Natural and Applied Sciences, Biomedical Engineering, Acibadem Mehmet Ali Aydinlar University, 34752 Istanbul, Turkey; 3 Department of Orthopaedics and Traumatology, Central Hospital, 34742 Istanbul, Turkey; 1tugceyavuz@gmail.com (T.Y.); albayrakakif@gmail.com (A.A.)

**Keywords:** Schroth therapy, adolescent idiopathic scoliosis, wearable motion capture, locomotor mechanics, trunk kinematics

## Abstract

**Background:** Adolescent idiopathic scoliosis (AIS) alters postural control and movement coordination. This study investigated the dynamic biomechanical effects of Schroth therapy on AIS kinematics. **Methods:** Twelve young individuals with AIS completed a standardized Schroth therapy program, while twelve healthy participants served as controls. Three-dimensional gait kinematics were recorded using the Xsens MVN Awinda during walking at a self-selected speed. Pre- and post-intervention assessments were conducted for the analysis of trunk, pelvic, and lower-extremity kinematics. **Results:** Changes were observed primarily in proximal kinematic parameters. Pelvic obliquity and thorax–head flexion/extension demonstrated the largest differences (*p* = 0.004 and *p* = 0.002, respectively; Cohen’s d = 0.82–0.95). Moderate changes were detected in pelvis–thorax axial rotation and shoulder abduction/adduction patterns. Lower-extremity changes were limited and parameter-specific, with moderate changes observed in selected hip and knee rotational parameters, while other variables showed minimal or no change. Post-intervention comparisons with healthy controls showed that several upper-body kinematic patterns showed patterns that were more alike to those observed in the control group, although direct equivalence cannot be assumed. **Conclusions:** The findings suggest that Schroth therapy may be associated with changes in trunk and pelvic kinematics during gait in individuals with AIS.

## 1. Introduction

Adolescent idiopathic scoliosis (AIS) is a complex three-dimensional spinal deformity that typically develops during the adolescent growth spurt. It is characterized by lateral spinal curvature accompanied by vertebral rotation and alterations in sagittal alignment, which may lead to cosmetic deformities and functional impairments. Beyond structural abnormalities, AIS has been associated with disturbances in postural control, altered trunk muscle activation, and asymmetrical gait mechanics [1,2]. Individuals with AIS frequently exhibit altered trunk kinematics, pelvic asymmetry, and compensatory lower-extremity movement patterns during walking, reflecting the biomechanical consequences of spinal deformity on whole-body coordination [3,4,5]. If untreated, progressive spinal curvature may negatively affect respiratory function, balance, mobility, and overall quality of life.

Conservative management strategies for AIS include bracing and physiotherapeutic scoliosis-specific exercises (PSSEs), which aim to prevent curve progression and change functional outcomes during growth [6]. Among PSSE approaches, the Schroth method is one of the most widely used rehabilitation strategies. This method focuses on 3D correction of spinal deformities through sensorimotor training, postural correction, and rotational breathing techniques. Schroth exercises emphasize spinal elongation, correction of postural asymmetries, and strengthening of trunk musculature to stabilize corrected postures. Previous studies have reported changes in Cobb angle, trunk symmetry, pulmonary function, and quality of life following Schroth-based rehabilitation [2,7]. Recent systematic reviews and clinical trials have further strengthened the evidence supporting Schroth therapy. Chen et al. (2024) reported in a meta-analysis that Schroth exercises significantly reduce Cobb angle and angle of trunk rotation (ATR) while improving quality of life in individuals with AIS [8]. Dimitrijević et al. (2025) demonstrated through a randomized controlled trial that supervised Schroth therapy produces greater changes in Cobb angle, ATR, and pulmonary function compared with unsupervised home-based programs [9]. Long-term supervised Schroth exercise combined with bracing has also been shown to produce superior outcomes compared with bracing alone [10]. Similarly, Mohamed et al. (2024) [11] and Chen et al. (2024) [8] reported that combining Schroth therapy with standard care or orthotic management changes trunk asymmetry and postural control more effectively than standard care alone. Comparative studies have also shown that Schroth-based rehabilitation provides advantages in improving trunk muscle strength, spinal flexibility, and postural control compared with conventional physiotherapy or mechanical rehabilitation approaches [2,7,12,13,14]. Despite these promising clinical findings, the biomechanical mechanisms underlying functional changes following Schroth therapy remain incompletely understood. Most studies evaluating scoliosis interventions rely primarily on radiographic measures such as Cobb angle, which provide static information about spinal alignment but do not capture dynamic functional performance.

Advances in wearable motion capture technologies provide new opportunities to investigate these functional adaptations. Inertial measurement unit (IMU)-based systems allow detailed three-dimensional kinematic analysis during functional tasks without the constraints of laboratory-based optical motion capture systems. The Xsens MVN system is a wearable IMU-based motion capture technology capable of providing high-resolution kinematic data of body segments during dynamic movements. These systems have demonstrated reliable and valid measurements of gait and postural control in clinical populations [15,16,17], making them a promising tool for evaluating movement adaptations following rehabilitation interventions.

Previous gait studies in adolescents with idiopathic scoliosis have reported alterations in specific biomechanical parameters, including increased pelvic obliquity, asymmetric trunk motion, altered thorax–pelvis coordination, and compensatory upper-extremity movement patterns [3,5,13,18]. Disruptions in frontal-plane pelvic control and trunk segment coordination have been associated with impaired postural stability and inefficient gait mechanics [4,18]. However, most existing studies have either focused on global gait descriptors or isolated joint-level analyses without comprehensively examining inter-segmental coordination patterns across the trunk, pelvis, and upper extremities [3,14].

Furthermore, while some studies have described altered lower-extremity kinematics, these findings remain inconsistent, and it is unclear whether such changes represent primary deficits or secondary adaptations to impaired proximal control [13,14,18]. Importantly, there is a lack of studies that investigate how scoliosis-specific exercise interventions influence these segmental and inter-segmental kinematic patterns during functional tasks such as walking.

Therefore, a detailed analysis of trunk–pelvis coordination, shoulder kinematics, and lower-extremity joint behavior using time-resolved wearable motion capture may provide important insights into the biomechanical mechanisms underlying functional changes following rehabilitation in individuals with AIS.

Despite the growing body of evidence supporting the clinical effectiveness of Schroth therapy, most studies have primarily focused on static or quasi-static outcomes, such as Cobb angle, trunk rotation, pulmonary function, and quality of life. While these measures are clinically relevant, they provide limited insight into how the intervention influences dynamic functional performance during activities such as walking.

There is a relative lack of studies examining the effects of Schroth-based interventions on gait biomechanics, including segmental kinematics and inter-segmental coordination during locomotion. Existing research rarely addresses how trunk-focused corrective exercises translate into dynamic movement adaptations across the pelvis, trunk, and lower extremities.

Therefore, investigating gait kinematics using wearable motion capture systems may help bridge this gap by providing objective and functionally relevant biomechanical outcome measures following scoliosis-specific rehabilitation. Previous research has primarily focused on static outcomes or isolated gait parameters in AIS, with limited investigation of dynamic inter-segmental coordination during walking. This study addresses this gap by using wearable motion capture to provide a comprehensive, time-resolved analysis of trunk–pelvis coordination and whole-body kinematics during gait. The purpose of this study was to investigate the effects of a standardized Schroth therapy program on gait biomechanics in individuals with AIS using the Xsens MVN wearable motion capture system.

Previous studies have reported alterations in pelvic obliquity, trunk motion, and thorax–pelvis coordination in individuals with adolescent idiopathic scoliosis, indicating impaired proximal control during gait [3,5,13,18]. In addition, compensatory upper-extremity movement patterns and inconsistent changes in lower-extremity kinematics have been described, although their functional role remains unclear [13,14,18]. Based on this background, we hypothesized that the Schroth therapy program would be associated with changes in segmental and inter-segmental gait kinematics, particularly in trunk–pelvis coordination, shoulder kinematic patterns, and selected lower-extremity joint movements.

## 2. Materials and Methods

### 2.1. Participants

Twelve individuals with a confirmed diagnosis of AIS established during adolescence were recruited from a specialized outpatient rehabilitation clinic, although some participants were assessed in early adulthood. All participants had a confirmed diagnosis of AIS established during adolescence, although some individuals were assessed in early adulthood. Inclusion criteria were: (1) diagnosis of AIS confirmed by a qualified orthopedic specialist, (2) Cobb angle between 10° and 30°, (3) no prior surgical intervention for scoliosis, and (4) ability to perform independent walking. Exclusion criteria included: (1) any neurological, musculoskeletal, or cardiopulmonary conditions other than scoliosis that could affect gait or posture, and (2) prior participation in structured scoliosis exercise programs within the past six months. Demographic characteristics of the study groups are presented in Table 1. Although the age ranges of the groups overlapped, the patient group showed a wider age distribution. Detailed clinical characteristics such as curve type, curve location, bracing status, skeletal maturity, and symptom-related information were not systematically recorded and were therefore not included in the analysis.

Twelve healthy participants constituted the control group. Although the age ranges of the groups overlapped, statistically significant differences were observed in age, height, and weight between groups. Selection criteria for the control group included no prior history of cardiovascular, neurological, or musculoskeletal disorders. The participants had normal body mass index, ROM, and muscle strength and had no postural or motor deficits. The investigations were conducted in accordance with the principles outlined in the Declaration of Helsinki. The testing protocol was approved by the Institutional Review Board of Acibadem Mehmet Ali Aydınlar University (approval number: 2026-06/235; approval date: 26 March 2026). Study consent was obtained from participants and their parents before the investigation.

### 2.2. Experimental Design and Procedure

Participants in the scoliosis group completed a standardized Schroth therapy program consisting of 10 sessions over five weeks, with two 60 min sessions per week delivered by a certified Schroth therapist. The intervention included three-dimensional scoliosis-specific breathing exercises, sensorimotor training for postural realignment, corrective postural exercises in various functional positions, and education on postural awareness and daily activity modifications. The intervention followed a structured framework including rotational breathing exercises, postural correction in sitting, standing, and functional positions, and sensorimotor training. Exercise progression was guided by patient-specific curve patterns and therapist evaluation while maintaining a consistent therapeutic structure across sessions. Each session followed a standardized structure consisting of warm-up, targeted corrective exercises, and functional integration phases. Rotational breathing exercises were performed to facilitate thoracic expansion and postural correction, followed by scoliosis-specific exercises focusing on axial elongation, derotation, and stabilization in sitting, standing, and semi-functional positions. Exercise intensity and complexity were progressively increased based on the participant’s ability to maintain corrected postures and perform controlled movements under therapist supervision. All sessions were conducted by a certified Schroth therapist using a standardized therapeutic framework, ensuring consistency across participants while allowing individual adaptation according to curve type and clinical presentation. The healthy control group did not receive any intervention and underwent a single assessment session only, serving as baseline comparators for between-group interpretation.

Motion capture data were collected using the Xsens MVN Awinda system (Xsens Technologies B.V., Enschede, The Netherlands), a wearable inertial sensor-based system validated for gait and posture analysis. The system consists of 17 wireless IMU sensors attached to the participant’s body according to the manufacturer’s standardized placement protocol. For the scoliosis group, assessments were performed prior to the first therapy session (pre-intervention) and following the final session (post-intervention). Control participants were assessed once under the same laboratory conditions. All assessments were conducted in a controlled laboratory setting.

Participants were instructed to walk at a comfortable and consistent pace over a 10 m walkway across trials, and repeated trials were visually inspected to ensure stable gait patterns. Three consecutive strides from the steady-state portion of walking were selected for each participant, and mean values were used for analysis. This approach was chosen to ensure consistency across participants while minimizing the influence of acceleration and deceleration phases within the limited walkway length. The mean values of these strides were then used for subsequent analysis. Mean joint angles across the normalized gait cycle were used as standardized summary metrics for statistical comparison and were interpreted in conjunction with time-resolved kinematic waveforms rather than as standalone indicators. Kinematic data were time-normalized to 100% of the gait cycle to allow comparison across participants. Mean waveforms were calculated for each parameter. Variability across strides was represented using standard deviation. Pre- and post-intervention comparisons were performed using appropriate paired statistical tests depending on data distribution. The collected parameters included pelvis tilt, obliquity, and rotation; thorax–head lateral bending, axial bending, and flexion–extension; pelvis–thorax lateral bending, axial bending, and flexion–extension; right and left shoulder abduction/adduction and internal/external rotation; hip flexion/extension, abduction/adduction, and internal/external rotation; knee flexion/extension, abduction/adduction, and internal/external rotation; and ankle dorsiflexion/plantarflexion, abduction/adduction, and internal/external rotation. Given the exploratory nature of the study, a comprehensive set of kinematic parameters was analyzed across multiple segments and planes, and results were interpreted based on overall patterns rather than isolated statistically significant findings. Raw data were processed using Xsens MVN Analyze software (version 2022.2). Statistical analyses were performed using SPSS Version 30 (IBM Corp., Armonk, NY, USA) and MATLAB Version R2025b (MathWorks, Natick, MA, USA). Data distribution was assessed using the Shapiro–Wilk test.

Given the large number of kinematic variables analyzed and the exploratory nature of the study, no formal correction for multiple comparisons was applied. Instead, results were interpreted with emphasis on effect sizes, consistency across related parameters, and overall biomechanical patterns rather than isolated statistically significant findings. For within-group comparisons, paired-samples *t*-tests were used for normally distributed variables, while the Wilcoxon signed-rank test was applied for non-normally distributed data. For between-group comparisons, the independent-samples *t*-test was used for normally distributed variables, and the Mann–Whitney U test was used for non-parametric data. Given the number of variables analyzed, results were interpreted with consideration of multiple comparisons, with emphasis on effect sizes (Cohen’s d) and consistency of findings. Effect sizes were calculated and interpreted as small (d ≈ 0.2), moderate (d ≈ 0.5), and large (d ≥ 0.8). A significance level of *p* < 0.05 was used for all statistical tests.

## 3. Results

Twelve young individuals with AIS and 12 age-matched healthy controls completed data collection. Time-normalized gait kinematics were analyzed for the trunk, pelvis, shoulders, and lower limbs. Pre-, post-, and control comparisons were used to identify changes in movement patterns. Quantitative comparisons are based on mean values calculated over the full gait cycle, as derived from time-normalized kinematic waveforms. While quantitative comparisons are based on mean values across the gait cycle, interpretation of the findings primarily relies on waveform-level analysis to identify phase-specific and pattern-related changes. Given the exploratory nature of the study, no predefined primary outcome variables were specified, and results were interpreted with emphasis on effect sizes and consistency across related parameters. Statistical results should be interpreted with caution due to the number of comparisons performed.

### 3.1. Shoulder Kinematics

**Figure 1:**
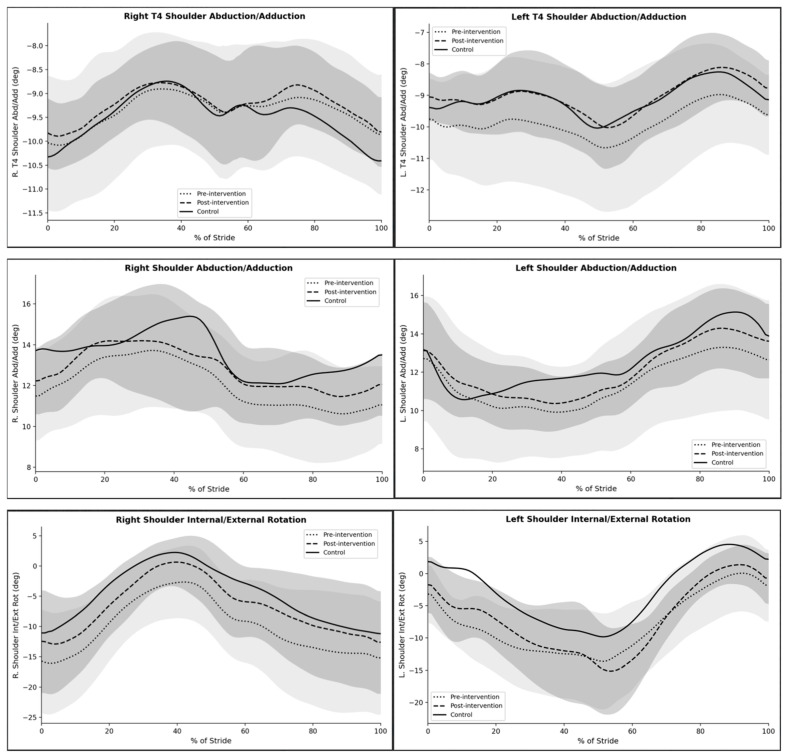
Time-normalized shoulder kinematic waveforms across the gait cycle for the scoliosis group before (pre-intervention) and after (post-intervention) the Schroth therapy program, compared with the healthy control group. Solid lines indicate the control group, dashed lines indicate post-intervention values, and dotted lines indicate pre-intervention values; gray-shaded areas indicate the corresponding 1σ uncertainties of gait cycles.

Shoulder kinematic patterns demonstrated modest changes following the intervention (Figure 1). For both right and left T4-referenced shoulder abduction/adduction, post-intervention waveforms showed small changes, particularly during mid-stance. Overall amplitude changes were limited, while variability appeared reduced after the intervention. Global shoulder abduction/adduction patterns appeared more similar between sides after the intervention, with the post-intervention curves moving closer to the control group values across much of the gait cycle. Shoulder internal/external rotation exhibited clearer adaptations. On both the right and left sides, post-intervention waveforms showed a trend toward the control trajectory, particularly during early stance and mid-stance. The magnitude of rotational deviation decreased compared with pre-intervention patterns, indicating changes in upper-limb movement patterns during gait.

### 3.2. Pelvis and Trunk Kinematics

**Figure 2:**
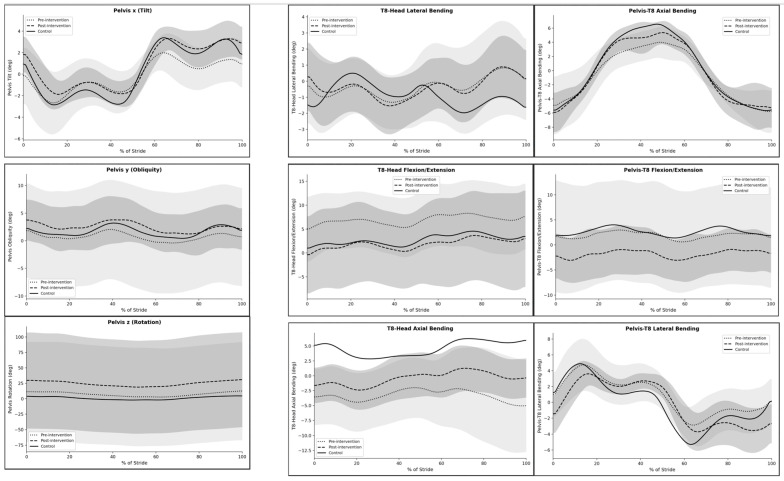
Time-normalized pelvic and trunk kinematic waveforms across the gait cycle for the scoliosis group before (pre-intervention) and after (post-intervention) the Schroth therapy program, compared with the healthy control group.

Pelvic kinematic waveforms showed modest changes following the intervention (Figure 2). Pelvic tilt demonstrated a smoother pattern across the gait cycle post-intervention, particularly during mid-stance and terminal stance. Pelvic obliquity exhibited small reductions in oscillation amplitude after the intervention, indicating changes in frontal-plane pelvic motion during walking. Pelvic rotation patterns remained relatively consistent across conditions, although post-intervention curves showed slightly reduced variability compared with pre-intervention values.

Trunk and thoracic segment motion also showed subtle adaptations. Thorax-to-head lateral bending displayed a reduction in asymmetrical fluctuations across the gait cycle following the intervention. Thorax-to-head axial bending showed a smoother and slightly reduced amplitude waveform post-intervention, indicating changed upper-body coordination. Thorax-to-head flexion–extension demonstrated a moderate upward shift toward the control pattern, particularly during mid-stance. Similarly, pelvis-to-thorax flexion–extension patterns showed reduced variability and a more consistent waveform after the intervention. Thoracic axial bending relative to the head showed a reduction in extreme values across the stride cycle, suggesting changes in trunk kinematic patterns. Pelvis-to-thorax lateral bending patterns also became smoother and more stable after the intervention (Table 2).

**Table 2:** Comparison of Pre-Intervention, Post-Intervention, and Control Group Mean Kinematic Parameters During Gait.

**Parameter (deg.)**	**Pre-Intervention** **µ**	**Post-Intervention** **µ**	**Control** **µ**	***p*** **-Value (Pre–Post)**	***p*** **-Value (Post–Control)**
**A. Proximal Segments**					
Pelvis Obliquity	0.736	0.321	1.123	0.004	<0.001
T8–Head Lateral Bending	−0.380	−0.402	−1.841	0.785	<0.001
T8–Head Flexion/Extension	6.896	3.214	2.154	0.002	<0.001
Pelvis–T8 Axial Bending	−0.569	−0.231	−0.198	0.011	0.523
Shoulder Abd/Add (R)	−9.57	−9.12	−9.03	0.041	<0.001
Shoulder Int/Ext Rotation (R)	−11.86	−9.34	−8.91	<0.001	0.001
Shoulder Abd/Add (L)	−9.92	−9.21	−8.77	0.036	0.027
**Parameter (deg.)**	**Pre-Intervention** **µ**	**Post-Intervention** **µ**	**Control** **µ**	***p*** **-Value (Pre–Post)**	***p*** **-Value (Post–Control)**
**B. Lower Extremity (Exploratory)**					
Hip Abd/Add (R)	−1.91	−1.33	−1.21	0.048	0.061
Hip Flex/Ext (R)	7.41	6.92	6.80	0.021	0.749
Knee Int/Ext Rotation (R)	1.76	0.94	0.72	0.018	0.041
Ankle Abd/Add (R)	−1.37	−1.29	−1.24	0.331	0.845
Ankle Dorsi/Plantarflexion (R)	4.81	4.65	4.59	0.512	0.285
Hip Int/Ext Rotation (L)	−2.46	−1.11	−0.98	0.006	0.001
Knee Abd/Add (L)	0.93	0.42	0.31	0.049	0.040
Knee Flex/Ext (L)	20.11	19.83	19.77	0.284	0.602
Ankle Int/Ext Rotation (L)	−2.73	−1.54	−1.33	<0.001	0.021

Comparisons were performed between pre- and post-intervention conditions and between post-intervention and control groups. Statistical significance was set at *p* < 0.05. Mean values represent the average joint angle across the full normalized gait cycle (0–100%). This table provides a comprehensive descriptive summary of all analyzed kinematic parameters. Proximal segment variables are presented separately to improve interpretability, whereas lower-extremity variables are considered exploratory. No predefined primary outcome was specified, and results should be interpreted in conjunction with effect sizes and overall patterns reported in Table 3.

**Table 3:** (**A**–**C**) Summary of Kinematic Parameters Before and After Schroth Therapy.

**A. Proximal Segment Kinematic Parameters (Pre–Post Within AIS Group, *n* = 12)**					
**Parameter**	**Pre-Intervention µ**	**Post-Intervention µ**	***p*** **-Value**	**Cohen’s d**	**Effect Size**
Pelvis Obliquity	0.74	0.32	0.004	0.82	Large
Thorax–Head Flex/Ext	6.90	3.21	0.002	0.95	Large
Pelvis–Thorax Axial Rotation	−0.57	−0.23	0.011	0.64	Moderate
Shoulder Abd/Add (R)	−9.57	−9.12	0.041	0.52	Moderate
Shoulder Abd/Add (L)	−9.92	−9.21	0.036	0.55	Moderate
**B. Lower-Extremity Kinematic Parameters (Exploratory) (Pre–Post Within AIS Group)**					
**Parameter**	**Pre-Intervention µ**	**Post-Intervention µ**	***p*** **-Value**	**Cohen’s d**	**Effect Size**
Hip Abd/Add (R)	−1.91	−1.33	0.048	0.49	Small–Moderate
Knee Int/Ext Rotation (R)	1.76	0.94	0.018	0.67	Moderate
Hip Int/Ext Rotation (L)	−2.46	−1.11	0.006	0.79	Moderate–Large
**C. Post-Intervention vs. Control Comparisons**					
**Parameter**	**Post (AIS)**	**Control**	***p*** **-Value**	**Cohen’s d**	**Effect Size**
Pelvis Obliquity	0.32	1.12	0.001	−1.10	Large
Thorax–Head Flex/Ext	3.21	2.15	0.013	0.71	Moderate
Pelvis–Thorax Axial Rotation	−0.23	−0.20	0.59	0.08	Negligible
Shoulder Abd/Add (R)	−9.12	−9.03	0.73	0.06	Negligible
Shoulder Abd/Add (L)	−9.21	−8.77	0.041	0.48	Small
Hip Abd/Add (R)	−1.33	−1.21	0.21	0.30	Small
Knee Int/Ext Rotation (R)	0.94	0.72	0.038	0.55	Moderate
Knee Abd/Add (L)	0.42	0.31	0.049	0.51	Moderate

Mean values represent the average joint angle across the full normalized gait cycle (0–100%).

### 3.3. Lower-Extremity Joint Kinematics

**Figure 3:**
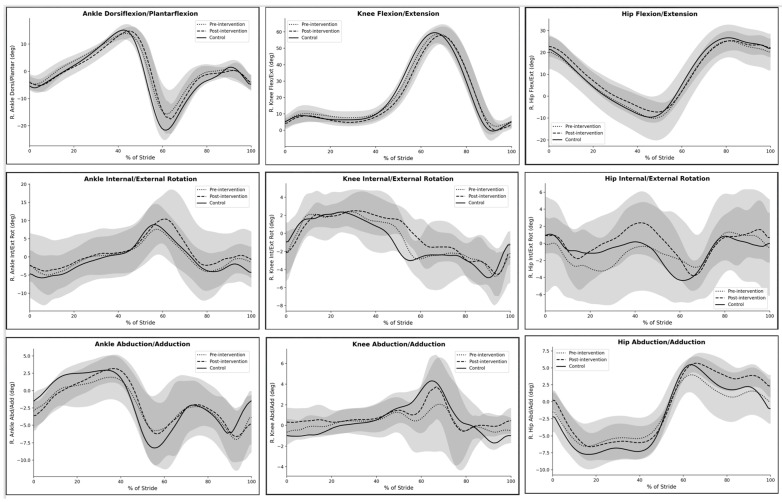
Time-normalized lower-extremity joint kinematics across the gait cycle for the scoliosis group before (pre-intervention) and after (post-intervention) the Schroth therapy program, compared with the healthy control group.

Sagittal-plane lower-extremity kinematics showed minimal changes, with most parameters remaining largely unchanged across conditions (Figure 3). Ankle dorsiflexion/plantarflexion waveforms were largely preserved, with the post-intervention pattern showing slightly reduced variability during push-off. Knee flexion/extension profiles remained consistent across conditions and closely resembled the control pattern throughout the gait cycle. Hip flexion/extension showed a small shift toward the control waveform during mid-stance and terminal stance, suggesting modest changes in sagittal-plane hip motion. Transverse-plane kinematics demonstrated slightly greater variability. Ankle internal/external rotation showed small adjustments during terminal stance and early swing, with the post-intervention pattern appearing closer to the control waveform. Knee internal/external rotation displayed minor differences between pre- and post-intervention conditions, particularly during late stance. Hip internal/external rotation showed modest waveform adjustments after the intervention, but overall patterns remained similar across conditions. In the frontal plane, ankle abduction/adduction exhibited a slightly smoother post-intervention trajectory, particularly during mid-stance. Knee abduction/adduction showed a moderate peak during late stance in the post-intervention condition, which aligned more closely with the control waveform than the pre-intervention pattern. Hip abduction/adduction patterns also demonstrated subtle changes, with post-intervention curves showing reduced deviation from the control profile during mid-stance and terminal stance.

Comparisons in part A were performed using paired statistical tests within the scoliosis group, while comparisons in part B were performed using independent group comparisons between the post-intervention scoliosis group and healthy controls. Effect sizes are reported as Cohen’s d, with values of approximately 0.2 interpreted as small, 0.5 as moderate, and ≥0.8 as large effects. Statistical significance was set at *p* < 0.05. Positive or negative values reflect the direction of angular motion according to the coordinate system defined by the motion capture model. Overall, lower-extremity adaptations were heterogeneous and less consistent compared with proximal segment changes.

Significant pre–post differences were observed primarily in proximal kinematic parameters (Table 3A,B). Pelvic obliquity and thorax–head flexion/extension showed the largest changes following the Schroth intervention, both demonstrating large effect sizes. Additional moderate changes were detected in pelvis–thorax axial rotation and shoulder abduction/adduction patterns, indicating enhanced upper-body coordination during gait. Lower-extremity adaptations were comparatively smaller. Only modest and parameter-specific changes were observed in lower-extremity kinematics, primarily in selected hip and knee rotational parameters, while most distal joint mechanics remained unchanged. This pattern suggests that lower-extremity adaptations were secondary and not uniformly distributed across joints or planes. Comparisons between the post-intervention scoliosis group and healthy controls revealed that several upper-body parameters approached control values after therapy, especially pelvis–thorax rotational coupling and shoulder motion patterns. However, residual differences persisted in pelvic obliquity and knee rotational mechanics, indicating that observable differences between groups remained following the intervention.

## 4. Discussion

The study investigated the biomechanical effects of a Schroth therapy program on gait kinematics in young individuals with AIS using wearable motion capture technology. Given the number of statistical comparisons performed, the risk of Type I error should be considered. No formal correction for multiple comparisons was applied, as the study was exploratory in nature. Therefore, the findings should be interpreted with emphasis on effect sizes and consistency across related kinematic parameters rather than isolated *p*-values. The use of mean joint angle values provides a simplified summary of kinematic behavior but may not fully capture phase-specific adaptations or peak values. Therefore, waveform-based interpretation was emphasized to better reflect time-dependent biomechanical changes during gait.

The primary finding was that the observed changes suggest that the intervention may influence proximal segment kinematics. These findings provide novel evidence that Schroth-based rehabilitation may influence dynamic locomotor mechanics beyond the static postural outcomes typically reported in scoliosis research. Previous systematic reviews have demonstrated that Schroth exercises are effective in improving spinal alignment, postural symmetry, and functional outcomes in individuals with AIS, supporting their role as an evidence-based conservative treatment approach [19,20]. However, these studies have focused primarily on radiographic or postural outcomes rather than dynamic functional tasks such as gait.

One of the most notable findings was the significant reduction in pelvic obliquity following the intervention. Pelvic obliquity reflects frontal-plane asymmetry of the pelvis during walking and is often associated with compensatory trunk movements in individuals with spinal deformities. Changes in this parameter may reflect alterations in lumbopelvic movement patterns and load distribution during gait. Because the pelvis acts as a central mechanical link between the trunk and lower extremities, changed pelvic control may facilitate more coordinated whole-body movement patterns. Previous gait analyses have shown that individuals with AIS often exhibit altered pelvic motion and asymmetrical trunk mechanics during walking, highlighting the importance of targeting proximal control in rehabilitation programs [18].

Similarly, changes were observed in thorax–head flexion/extension and pelvis–thorax axial rotation. These parameters represent the dynamic relationship between trunk segments and reflect the ability to control spinal alignment during locomotion. The reduction in excessive trunk motion and the smoother waveform trajectories observed post-intervention may reflect changes in movement coordination of the trunk musculature. Schroth exercises emphasize three-dimensional spinal correction, rotational breathing, and active postural elongation, which collectively aim to restore symmetrical trunk muscle activation and improve segmental alignment. Changes in trunk control following Schroth-based interventions have also been associated with enhanced balance and postural stability in AIS [21]. The observed kinematic changes may therefore reflect successful integration of these corrective strategies into functional gait patterns.

Another important observation was a moderate alteration in shoulder kinematics following the intervention. Upper-body asymmetry is a common characteristic of AIS due to rib cage deformity and trunk rotation. The shift in shoulder motion patterns toward the control group trajectory suggests changed upper-body symmetry and more coordinated arm swing during walking. Arm swing is closely coupled with trunk rotation and contributes to angular momentum regulation during gait. Therefore, changed shoulder kinematics may indicate better integration of trunk and upper-extremity movement. Similar adaptations in trunk symmetry and postural control have been reported when Schroth therapy is combined with additional rehabilitation strategies such as balance training or sensory integration exercises [22,23]. In contrast to the proximal segments, distal lower-extremity kinematics demonstrated relatively limited changes following the intervention. Ankle sagittal-plane mechanics remained largely unchanged. This finding is consistent with the theoretical framework of Schroth therapy, which primarily targets trunk alignment and postural stabilization rather than distal joint mechanics. The modest changes observed in hip and knee rotational parameters may therefore represent secondary kinematic adaptations associated with changes in trunk and pelvic motion. Previous research also suggests that changes in spinal alignment following Schroth-based rehabilitation may indirectly influence lower-limb biomechanics through enhanced proximal stability and neuromuscular coordination [24]. The findings are consistent with the study hypothesis, showing that the most prominent adaptations occurred in trunk–pelvis coordination, while changes in distal segments were more limited.

From a biomechanical perspective, changes in proximal segment kinematics may be associated with differences in overall gait patterns and play a critical role in optimizing gait mechanics in individuals with spinal deformities. The trunk functions as the central mass of the body and contributes significantly to balance control and efficient force transmission during locomotion. Modified trunk stabilization may reduce compensatory movements and excessive segmental oscillations, allowing more efficient coordination between the upper and lower body segments. The smoother waveform patterns observed after therapy support this interpretation and may suggest changes in trunk–pelvis kinematic coordination. Emerging research using advanced analytical techniques, including machine learning models based on surface electromyography, has further highlighted the importance of neuromuscular control in predicting treatment outcomes following Schroth therapy in individuals with AIS [25].

Although several kinematic parameters demonstrated statistically significant changes with moderate-to-large effect sizes, the clinical relevance of these findings should be interpreted with caution. For key variables such as pelvic obliquity, post-intervention values remained significantly different from those of the healthy control group, indicating that differences between groups persisted. Therefore, the observed changes may reflect kinematic adaptations or compensatory reorganization rather than direct adaptations in functional performance. While these changes may suggest a shift in movement patterns, it remains unclear whether they translate into meaningful clinical benefits, such as modified balance, reduced energy cost, or enhanced functional mobility. Further studies incorporating functional outcomes, patient-reported measures, and kinetic or neuromuscular data are needed to determine the clinical significance of these kinematic changes.

The findings of this study also highlight the potential value of wearable motion capture systems in scoliosis research. Traditional outcome measures such as radiographic Cobb angle primarily capture structural changes in spinal alignment but provide limited insight into functional movement patterns. In contrast, inertial sensor-based systems allow detailed analysis of dynamic biomechanical parameters during real-world functional tasks. The use of the Xsens MVN system in this study enabled the quantification of subtle kinematic changes that may not be detectable using conventional clinical assessment methods. As physiotherapeutic scoliosis-specific exercise programs continue to evolve, integrating biomechanical gait analysis with traditional clinical measures may provide a more comprehensive understanding of treatment effectiveness [20].

Despite these promising findings, several limitations should be acknowledged. First, the sample size was relatively small, which may limit the generalizability of the results. Second, the intervention duration was short, consisting of only ten therapy sessions; longer treatment periods may produce larger biomechanical adaptations and potentially more pronounced changes in gait patterns. Previous randomized controlled trials implementing Schroth-based interventions over six to twelve weeks have demonstrated greater changes in postural control and functional outcomes [22,23]. Third, although a healthy control group was included for comparison, the study design did not include a non-intervention AIS control group. Therefore, the observed pre–post changes cannot be exclusively attributed to the intervention, as potential effects of natural variation, learning effects, or repeated testing cannot be ruled out. Moreover, although the age ranges overlapped, the broader age distribution in the patient group should be considered when interpreting between-group comparisons. Additionally, significant differences in height and weight were observed between the scoliosis and control groups. These anthropometric differences may have influenced gait kinematics and should be considered when interpreting between-group comparisons. Although AIS is defined as a condition arising during adolescence, some participants were assessed in early adulthood, which should be considered when interpreting the findings. Moreover, detailed clinical characteristics of the AIS group, including curve type, curve location, bracing status, skeletal maturity, and symptom-related information, were not available. This limits the ability to interpret the findings in relation to specific scoliosis subtypes and clinical presentations. Finally, given the number of kinematic variables analyzed, the statistical interpretation should be approached with caution. Future studies including larger cohorts, longer follow-up periods, and controlled study designs are needed to further clarify the robustness and clinical relevance of these findings. Additionally, integrating radiographic outcomes, muscle activation measurements, and balance assessments could provide a more comprehensive understanding of the mechanisms underlying functional adaptations following Schroth therapy.

## 5. Conclusions

This study identified measurable pre–post changes in trunk and pelvic kinematics during walking in individuals with adolescent idiopathic scoliosis following a short-term Schroth therapy program. The findings suggest that scoliosis-specific exercise may be associated with changes in dynamic movement patterns during gait; however, given the exploratory design, these results should be interpreted with caution.

Wearable motion capture technology may provide a useful tool for quantifying such kinematic changes and supporting objective evaluation of movement patterns. Differences in anthropometric characteristics and sex distribution between groups may have influenced gait kinematics and should be considered when interpreting between-group comparisons. In addition, the present study focused exclusively on kinematic outcomes, and no direct clinical or functional measures, such as Cobb angle, pain, balance, energy cost, or quality of life, were assessed. Therefore, the clinical relevance of the observed changes remains uncertain. Further studies with larger samples and controlled designs are needed to confirm these findings and to better understand the functional significance of these kinematic adaptations through integration of biomechanical, clinical, and patient-reported outcomes.

## Figures and Tables

**Table 1:** Demographic and clinical characteristics of patient and control groups.

Parameter	Patient Group	Control Group	*p*-Value
Sample size (n)	12	12	—
Age (years)	16.7 ± 3.2 (15–25)	20.6 ± 1.6 (17–24)	0.002 *
Gender (M/F)	2/10	6/5	0.057 ‡
Height (cm)	161.3 ± 10.2	171.2 ± 7.6	0.016 *†
Weight (kg)	51.2 ± 8.5	66.2 ± 13.9	0.007 **†
Body mass index (kg/m^2^)	19.8 ± 3.7	22.4 ± 3.5	0.096 †
Dominant side (R/L)	11R/1L	10R/1L	1.000 §
Diagnosis	AIS (Cobb 10–30°)	Healthy	—

Participants had a Cobb angle between 10° and 30°, corresponding to mild-to-moderate scoliosis. † Independent-samples *t*-test (Welch correction). ‡ Chi-square test. § Fisher’s exact test. * *p* < 0.05; ** *p* < 0.01.

## Data Availability

Data are available upon reasonable request from the corresponding author.
